# Linking peripheral atherosclerosis to blood–brain barrier disruption: elucidating its role as a manifestation of cerebral small vessel disease in vascular cognitive impairment

**DOI:** 10.1007/s11357-024-01194-0

**Published:** 2024-06-03

**Authors:** Ádám Nyúl-Tóth, Roland Patai, Anna Csiszar, Anna Ungvari, Rafal Gulej, Peter Mukli, Andriy Yabluchanskiy, Zoltan Benyo, Peter Sotonyi, Calin I. Prodan, Eric M. Liotta, Peter Toth, Fanny Elahi, Péter Barsi, Pál Maurovich-Horvat, Farzaneh A. Sorond, Stefano Tarantini, Zoltan Ungvari

**Affiliations:** 1 Vascular Cognitive Impairment, Neurodegeneration and Healthy Brain Aging Program, Department of Neurosurgery, University of Oklahoma Health Sciences Center, Oklahoma City, OK USA; 2 Oklahoma Center for Geroscience and Healthy Brain Aging, University of Oklahoma Health Sciences Center, Oklahoma City, OK USA; 3 Department of Public Health, Semmelweis University, Semmelweis University, Budapest, Hungary; 4 Stephenson Cancer Center, University of Oklahoma, Oklahoma City, OK USA; 5 Department of Health Promotion Sciences, College of Public Health, University of Oklahoma Health Sciences Center, Oklahoma City, OK USA; 6 Doctoral College/Department of Public Health, International Training Program in Geroscience, Semmelweis University, Budapest, Hungary; 7 Institute of Translational Medicine, Semmelweis University, 1094 Budapest, Hungary; 8 Cerebrovascular and Neurocognitive Disorders Research Group, HUN-REN, Semmelweis University, 1094 Budapest, Hungary; 9 Department of Vascular and Endovascular Surgery, Heart and Vascular Centre, Semmelweis University, 1122 Budapest, Hungary; 10 Veterans Affairs Medical Center, Oklahoma City, OK USA; 11 Department of Neurology, University of Oklahoma Health Sciences Center, Oklahoma City, OK USA; 12 Department of Neurology, Division of Stroke and Neurocritical Care, Northwestern University Feinberg School of Medicine, Chicago, IL USA; 13 Department of Neurosurgery, Medical School, University of Pecs, Pecs, Hungary; 14 Neurotrauma Research Group, Szentagothai Research Centre, University of Pecs, Pecs, Hungary; 15 ELKH-PTE Clinical Neuroscience MR Research Group, University of Pecs, Pecs, Hungary; 16 Departments of Neurology and Neuroscience Ronald M. Loeb Center for Alzheimer’s Disease Friedman Brain Institute Icahn School of Medicine at Mount Sinai, New York, NY USA; 17 James J. Peters VA Medical Center, Bronx, NY USA; 18 ELKH-SE Cardiovascular Imaging Research Group, Department of Radiology, Medical Imaging Centre, Semmelweis University, Budapest, Hungary

**Keywords:** Aging, Atherosclerosis, Atherogenesis, Cerebral circulation, Cerebromicrovascular, White matter hyperintensities, Large vessel disease, Senescence, Arteriosclerosis, Peripheral artery disease, White matter injury, White matter hyperintensities, Leukoaraiosis

## Abstract

Aging plays a pivotal role in the pathogenesis of cerebral small vessel disease (CSVD), contributing to the onset and progression of vascular cognitive impairment and dementia (VCID). In older adults, CSVD often leads to significant pathological outcomes, including blood–brain barrier (BBB) disruption, which in turn triggers neuroinflammation and white matter damage. This damage is frequently observed as white matter hyperintensities (WMHs) in neuroimaging studies. There is mounting evidence that older adults with atherosclerotic vascular diseases, such as peripheral artery disease, ischemic heart disease, and carotid artery stenosis, face a heightened risk of developing CSVD and VCID. This review explores the complex relationship between peripheral atherosclerosis, the pathogenesis of CSVD, and BBB disruption. It explores the continuum of vascular aging, emphasizing the shared pathomechanisms that underlie atherosclerosis in large arteries and BBB disruption in the cerebral microcirculation, exacerbating both CSVD and VCID. By reviewing current evidence, this paper discusses the impact of endothelial dysfunction, cellular senescence, inflammation, and oxidative stress on vascular and neurovascular health. This review aims to enhance understanding of these complex interactions and advocate for integrated approaches to manage vascular health, thereby mitigating the risk and progression of CSVD and VCID.

## Introduction

Cerebral small vessel disease (CSVD) emerges as a critical yet frequently underappreciated component within the complex landscape of age-related neurovascular disorders [1–5]. This multifaceted spectrum involves a range of pathologies that affect the cerebral microcirculation, including small arteries, arterioles, capillaries, and postcapillary venules, and plays a substantial role in stroke and cognitive impairment and dementia associated with aging [2, 5–9]. CSVD stands as a key factor in the emergence and progression of vascular cognitive impairment and dementia (VCID) and contributes notably to the pathogenesis of dementias within the Alzheimer’s disease (AD) spectrum [2, 5, 7–12]. Neuropathologically, CSVD includes a spectrum of pathologies impacting perforating arteries, arterioles, capillaries, and veins in the brain, as well as the leptomeningeal vessels [6]. Histologically, it is characterized by arteriolosclerosis, lipohyalinosis, fibrinoid necrosis, and cerebral amyloid angiopathy (CAA), among other histopathological categorizations [6].

**Fig. 1:**
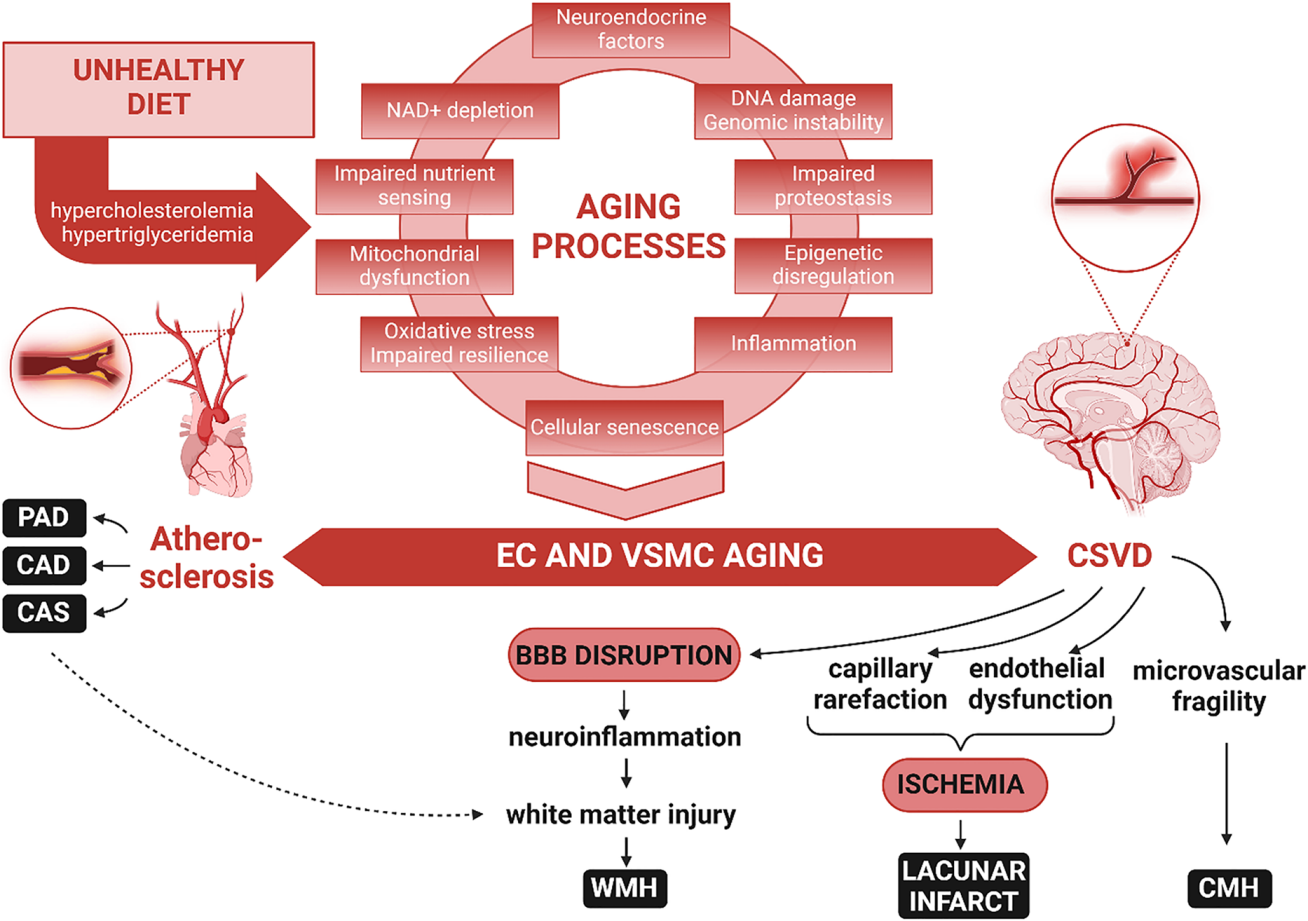
Bridging atherosclerosis and CSVD: unraveling the continuum of accelerated vascular aging. This figure presents a conceptual model illustrating how fundamental cellular and molecular aging mechanisms synergistically drive the progression of both macrovascular and microvascular aging. The upper section of the figure delineates the interconnected aging hallmarks, such as oxidative stress, mitochondrial dysfunction, cellular senescence, and increased inflammation. These aging processes synergistically induce functional and phenotypic alterations in endothelial cells (ECs) and vascular smooth muscle cells (VSMCs), laying the groundwork for various aging-associated vascular diseases. Lifestyle risk factors, notably unhealthy diets, exacerbate these vascular aging pathways, leading to the development of atherosclerosis in large arteries, manifesting as carotid artery stenosis (CAS), coronary artery disease (CAD), and peripheral artery disease (PAD), and CSVD within the cerebral microcirculation. The diagram proposes that atherosclerotic vascular diseases and CSVD share common aging origins, explaining their frequent co-occurrence in the elderly, including manifestations such as cerebral microhemorrhages (CMHs), lacunar infarcts, and white matter hyperintensities (WMHs). Aging-induced dysfunction of cerebromicrovascular endothelial cells culminates in BBB disruption, fostering neuroinflammation. This, alongside regional ischemia from reduced cerebral blood flow (CBF) due to capillary rarefaction and endothelial dysfunction, contributes to the development of WMHs

Functionally, CSVD leads to several critical consequences, such as endothelial dysfunction and cerebral blood flow dysregulation, leading to brain ischemia [8, 13–17]; increased microvascular fragility resulting in cerebral microhemorrhages (CMHs) [18]; and blood–brain barrier (BBB) disruption [19–23], which triggers neuroinflammation [24, 25]. Among these, the disruption of the BBB is particularly pivotal [24–27], serving as the focal point of this review. The BBB is a critical regulator of the cerebral microenvironment, that ensures the protection of neural tissue from systemic influences. Disruption of the BBB is a key feature of CSVD and contributes significantly to increased neuroinflammation, demyelination, impaired synaptic communication, neuronal damage, and cognitive decline [24, 25] (Fig. 1).

The aforementioned pathophysiological alterations of the cerebral microcirculation underpin the imaging signs of CSVD [1, 3, 28–30], which are crucial for its clinical diagnosis. These include white matter hyperintensities (WMHs), CMHs, enlarged perivascular spaces, and lacunar infarcts [1, 3, 28–32], with WMHs playing a particularly significant role as their pathogenesis involves BBB disruption [26, 33–40] (Fig. 1). Understanding CSVD, an age-related disease, necessitates a deep dive into the mechanisms driving and accelerating cerebromicrovascular aging, especially how this aging process promotes BBB disruption [24, 25, 41, 42].

Vascular aging represents a comprehensive, multifaceted process that affects the vascular system as a whole, ranging from the large arteries to the microvasculature [41, 42]. It is understood not as a series of isolated occurrences within various vessel sizes but as a continuum of interrelated changes across the vascular network [41–43]. In this context, the intricate link between systemic cardiovascular health and accelerated, premature development of age-related microvascular pathologies (“accelerated cerebromicrovascular aging”) becomes evident [14, 36, 44–49]. The age-associated pathological alterations in larger vessels, such as those seen in atherosclerosis, are fundamentally connected to changes in the microvasculature, contributing to CSVD development [44–49]. The risk factors known to accelerate cellular aging processes and thereby atherogenesis in larger arteries also play a role in accelerating microvascular aging, thus promoting CSVD [41, 42]. Risk factors accelerating microvascular aging are also known to promote BBB disruption [24, 50–56]. Here, the concept of the continuum of accelerated vascular aging, linking atherosclerosis—an age-related disease of larger vessels—to CSVD, is crucial [41, 44–49].

This review aims to synthesize the current knowledge and recent advancements in understanding the connection between peripheral atherosclerosis and BBB disruption and how this interaction contributes to CSVD and VCID. We will explore the mechanisms by which atherosclerosis may affect BBB integrity and examine the clinical implications of this relationship. Furthermore, this review will highlight emerging research, potential therapeutic targets, and future research directions. By providing a comprehensive overview of these interconnected pathways, we aim to deepen the understanding of the systemic nature of neurovascular disorders and contribute to the development of more effective prevention and treatment strategies.

## CSVD, white mater damage, and VCID

### Neuroimaging techniques and their role in diagnosis

Neuroimaging plays a crucial role in the diagnosis and management of CSVD and VCID [30, 57–59]. Computed tomography (CT) can provide a basic impression but magnetic resonance imaging (MRI) is the method of choice in identifying hallmark features of CSVD, including WMHs, lacunar infarcts, CMHs, lobar hemorrhages, superficial siderosis, perivascular space (PVS) enlargement, and cerebral atrophy [30, 57–59].

WMHs are commonly detected through advanced neuroimaging techniques. MRI has emerged as the gold standard for identifying WMHs. T2-weighted sequences are highly sensitive to changes in water content while fluid-attenuated inversion recovery (FLAIR) imaging can effectively differentiate WMHs with gliosis from the surrounding normal brain tissue and from PVSs by suppressing the signal from cerebrospinal fluid, enhancing the contrast and visibility of these lesions [30, 57–59]. CMHs and their specific locations play a crucial role in the differential diagnosis of CSVD, particularly in distinguishing between the most common sporadic types, such as arteriolosclerosis induced by hypertension, and cerebral amyloid angiopathy. While CT scans can readily identify larger lobar hemorrhages or those occurring in the basal ganglia and thalami, CMHs and superficial siderosis remain undetectable on both CT and standard MRI sequences. Their detection is reliant on hemosiderin-sensitive MRI sequences, such as gradient echo (GRE) T2* or, more effectively, susceptibility weighted imaging (SWI), which are specialized for identifying the hemosiderin deposits indicative of past bleeding events. Diffusion weighted imaging (DWI) is the primary tool to detect cytotoxic oedema resulting from recent ischemic events. Additionally, diffusion tensor imaging (DTI) provides insights into the microstructural integrity of white matter, offering complementary information about the extent and nature of WMHs. Advanced MRI techniques, such as functional MRI (fMRI), shed light on the functional connectivity of brain networks, offering further understanding of white matter integrity and the potential impact of WMHs on brain networks.

The severity of CSVD can be quantitatively assessed using MRI [30, 58–65]. The Fazekas grading system classifies CSVD into mild (grade 1), moderate (grade 2), and severe (grade 3) categories, based on the quantity, appearance, and distribution of WMHs. Additional diagnostic considerations include evaluating the extent of hyperintensities and lacunar infarcts that affect critical regions such as the basal ganglia, thalami, pons, and cerebellum. Furthermore, the number and spatial distribution of CMHs play a crucial role in determining the overall severity of CSVD, offering a comprehensive view of its impact.

Another significant capability of MRI is the detection of cerebral atrophy, allowing for the assessment of brain volume reduction. Visual grading scales serve as valuable tools in basic diagnostic procedures, while more detailed scientific evaluations rely on volumetric analyses or voxel-based morphometry, utilizing three-dimensional (3D) GRE T1 sequences. These advanced techniques offer precise measurements of the volumes of grey matter, white matter, and cerebrospinal fluid (CSF) compartments across various brain regions. By comparing data from follow-up MRIs, the rate of neurodegenerative processes can be accurately determined. Additionally, employing data from 3D FLAIR sequences, these methods can also quantify the extent of WMHs and track their progression over time, providing comprehensive insights into cerebral changes.

These imaging modalities not only facilitate the diagnosis of CSVD but also aid in understanding its progression and impact on cognitive function, thus serving as critical tools in the clinical assessment of VCID and in guiding therapeutic interventions directed at modifying underlying vascular risk factors.

### Epidemiology

The prevalence of CSVD varies widely depending on the studied population, the diagnostic criteria used, and the sensitivity of the imaging techniques [66]. In general, CSVD is more common in older adults, with studies suggesting that signs of CSVD can be found in more than 90% of older adults when using sensitive MRI criteria [66]. Recent studies utilizing advanced imaging techniques suggest that around 50% of the older general population may exhibit CMHs [2, 67–70]. The occurrence of WMHs, both subcortical and periventricular, is more commonly observed in individuals over the age of 60, with their prevalence notably increasing with advancing age [66]. Data from the Rotterdam Scan Study highlighted that the prevalence of subcortical and periventricular WMHs rose by 0.2% and 0.4%, respectively, for each additional year of age [66]. Specifically, among those aged 60 to 70 years, 87% presented with subcortical WMHs and 68% with periventricular WMHs [66]. The figures escalated to 100% for subcortical and 95% for periventricular WMHs among individuals aged 80 to 90 years [66]. The prevalence also increases with risk factors such as hypertension, diabetes mellitus, smoking, and hyperlipidemia [66, 71, 72]. CSVD is increasingly recognized as a major contributor to age-related cognitive decline and VCID [71]. VCID, which ranges from mild cognitive impairment to fully developed vascular dementia, is believed to affect a considerable percentage of the elderly, though precise figures vary due to differences in diagnostic criteria [71]. These conditions not only impose a substantial burden on individuals and healthcare systems but also highlight the importance of early detection and intervention.

### Pathogenesis of CSVD: role of white matter injury

The pathogenesis of CSVD and VCID is intricately linked to white matter injury, which plays a pivotal role in the disease’s progression and its clinical manifestations [13, 17, 26, 35, 71, 73–79] (Fig. 1). White matter injury in CSVD is primarily characterized by demyelination, axonal loss, and disruptions in white matter tract integrity, consequences of chronic hypoperfusion and BBB breakdown [26, 34, 35, 37, 38, 40]. These pathological changes result in impaired white matter connectivity, affecting the brain's ability to communicate effectively across different regions. The disruption of these neural pathways is a key contributor to the clinical symptoms of CSVD, including cognitive decline, gait disturbances, and mood changes [26, 35, 71, 74, 79–91]. Understanding the mechanisms underlying white matter injury in CSVD is crucial for developing targeted therapeutic strategies aimed at preserving white matter integrity and preventing disease progression. The pathogenesis of CSVD is complex and multifactorial, involving accelerated cellular and molecular mechanisms of aging, modulated by genetic, lifestyle, and environmental factors [8, 9, 33, 92].

## BBB disruption as a manifestation of CSVD

### Role in the pathogenesis of VCID

BBB disruption is a prominent manifestation of CSVD [24, 25] and plays a significant role in the pathogenesis of VCID [21, 93]. The BBB, a selective barrier crucial for maintaining cerebral homeostasis, when compromised, leads to an influx of neurotoxic substances, proteins, cytokines, metabolites, and bacterial breakdown products into the brain parenchyma [24]. BBB disruption is thought to contribute to microglia activation, neuroinflammation, neuronal, and synaptic dysfunction and white matter injury, all characteristic features of VCID [5, 24, 25, 32, 36, 43, 57, 94–96]. The resulting damage is closely linked to the cognitive decline seen in VCID, illustrating the critical role of BBB integrity in preserving cognitive function [5, 24, 25, 32, 36, 43, 57, 94–96]. Additionally, the interplay among CSVD, BBB disruption, and neurodegenerative processes, particularly in the context of Alzheimer’s disease, adds another layer of complexity to the pathogenesis of VCID [24, 25].

In CSVD, BBB impairment is often attributed to endothelial cell dysfunction and age-related pathological changes in the cerebral microcirculation, often exacerbated by chronic hypertension [5, 8, 33, 36, 38, 69, 97–99], obesity, and/or metabolic diseases [51, 100–104]. Preclinical research further illuminates this understanding, showing that hallmarks of aging, such as endothelial senescence, increased presence of pro-inflammatory and pro-geronic circulating factors, and a decline in anti-geronic humoral factors contribute significantly to BBB disruption [43, 105–111]. These insights have been particularly highlighted by heterochronic parabiosis experiments and studies on transgenic animals, which demonstrate how aging-related systemic factors can influence the integrity of the BBB, thereby exacerbating the pathophysiological processes of CSVD and VCID [112–114].

### Biomarkers for BBB dysfunction in CSVD

Detecting BBB dysfunction in CSVD is challenging, but given the importance of this multi-cellular phenotype in understanding connections between CSVD and brain degeneration, efforts have led to the development of several novel imaging approaches [32, 115, 116] and quantitative biofluid measures, such as CSF/plasma, CSF/serum albumin quotient, and CSF fibrinogen [26, 117–121]. Dynamic contrast-enhanced MRI (DCE-MRI) and dynamic susceptibility contrast MRI (DSC-MRI) capture leakage of BBB based on modeling of gadolinium entry into the brain parenchyma. Additional MRI methods, such as arterial spin labeling (ASL MRI), do not require contrast agents and measure cerebral blood flow [122]. Together, DCE-MRI and ASL MRI provide complementary perspectives on CSVD. This combination of techniques enhances the accuracy and depth of understanding of CSVD, perfusion and BBB integrity, crucial for diagnosing and monitoring neurological diseases that compromise BBB function. Beyond imaging, biomarkers in cerebrospinal fluid [23], and circulating markers of endothelial dysfunction and BBB permeability are under development [123–128]. Examples include CSF/plasma albumin ratio [117, 118], CSF fibrinogen levels [129], levels of matrix metallopeptidases 2 and 9 (MMP2 and MMP9) in serum and CSF [130–132], and soluble platelet-derived growth factor receptor beta (sPDGFRβ) content in CSF [133–135]. The combination of biomarkers of CSVD and BBB dysfunction and plasma and CSF proteomics will shed light on drivers of barrier dysfunction and mediators of CSVD-related brain degeneration in individuals harboring CSVD.

## Continuum of vascular aging: peripheral atherosclerosis and its impact on the brain

The continuum of vascular aging presents a comprehensive framework that connects aging-associated pathophysiological alterations in large peripheral arteries, like those seen in atherosclerosis, with changes in the microvasculature that culminate in CSVD and BBB disruption [8, 41–43, 94, 109, 136, 137] (Fig. 1). This holistic perspective underscores the necessity of considering vascular health as an integrated system, where macrovascular and microvascular pathologies interact synergistically. A wealth of translational, experimental, and clinical evidence supports the linkage between peripheral atherosclerosis and CSVD, highlighting their collective impact on VCID as well as Alzheimer’s disease [138, 139]. This interconnectedness emphasizes the importance of a unified approach to understanding and addressing vascular aging and its implications for brain health.

### Clinical evidence linking atherosclerosis to CSVD

**Fig. 2:**
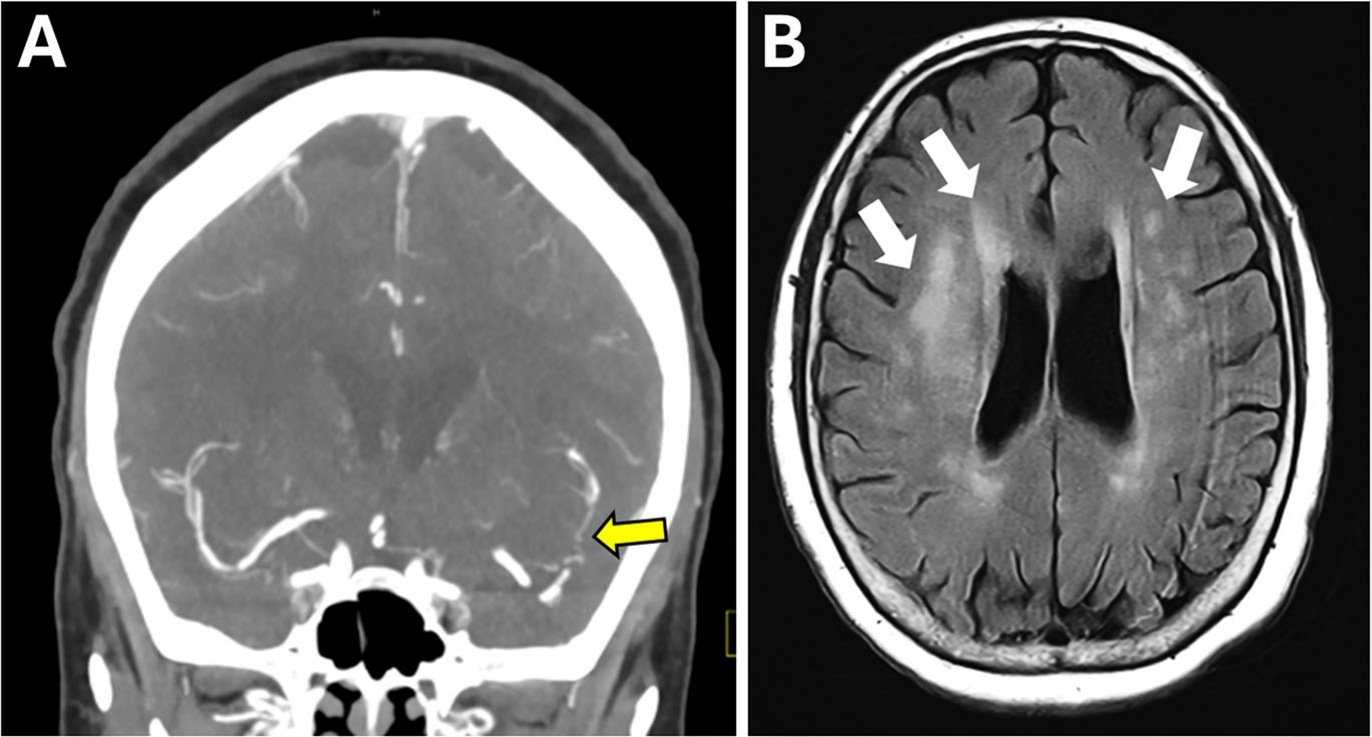
A case of converging pathways: atherosclerosis and CSVD in a senior patient. This figure presents MRI findings from a 77-year-old female with a history of hypertension, insulin-dependent diabetes mellitus, and Crohn’s disease, who exhibited symptoms of weakness and disorientation. CT angiography revealed severe stenosis of the proximal right middle cerebral artery (MCA) and its M2 branches (panel **A**, arrow), indicative of intracranial atherosclerosis. Concurrently, MRI FLAIR images showcased white matter hyperintensities (panel **B**, arrows), aligning with a diagnosis of moderate severity chronic CSVD, alongside an acute infarct in the right MCA watershed territory (not shown). This case vividly illustrates the interconnected nature of vascular pathologies, encapsulating the essence of accelerated vascular aging's impact on both large and small cerebral vessels

**Fig. 3:**
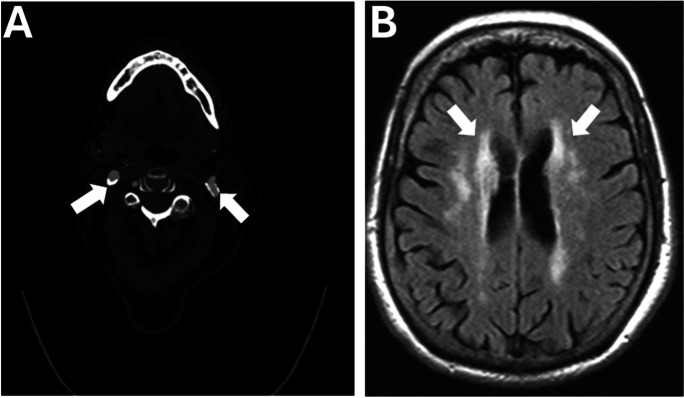
Vascular aging in full spectrum: a complex case of multisystem involvement. This figure features the case of an 87-year-old female with a comprehensive medical history, including asthma, insulin-dependent diabetes mellitus, hypertension, hyperlipidemia, atherosclerotic coronary artery disease, and stage 3 chronic kidney disease, who presented with abdominal pain and subsequently experienced transient slurred speech and right facial numbness. CT angiography highlighted calcified atherosclerotic plaques in the bulbs of the bilateral internal carotid arteries (**A**, arrows), illustrating the widespread impact of systemic atherosclerosis. MRI FLAIR images revealed white matter hyperintensities (**B**, arrows), suggestive of chronic CSVD, without evidence of acute ischemia, leading to a diagnosis of a transient ischemic attack. This case underscores the multifaceted nature of vascular aging, showcasing how systemic atherosclerotic changes and small vessel disease converge, affecting both cerebral and peripheral vascular health

**Fig. 4:**
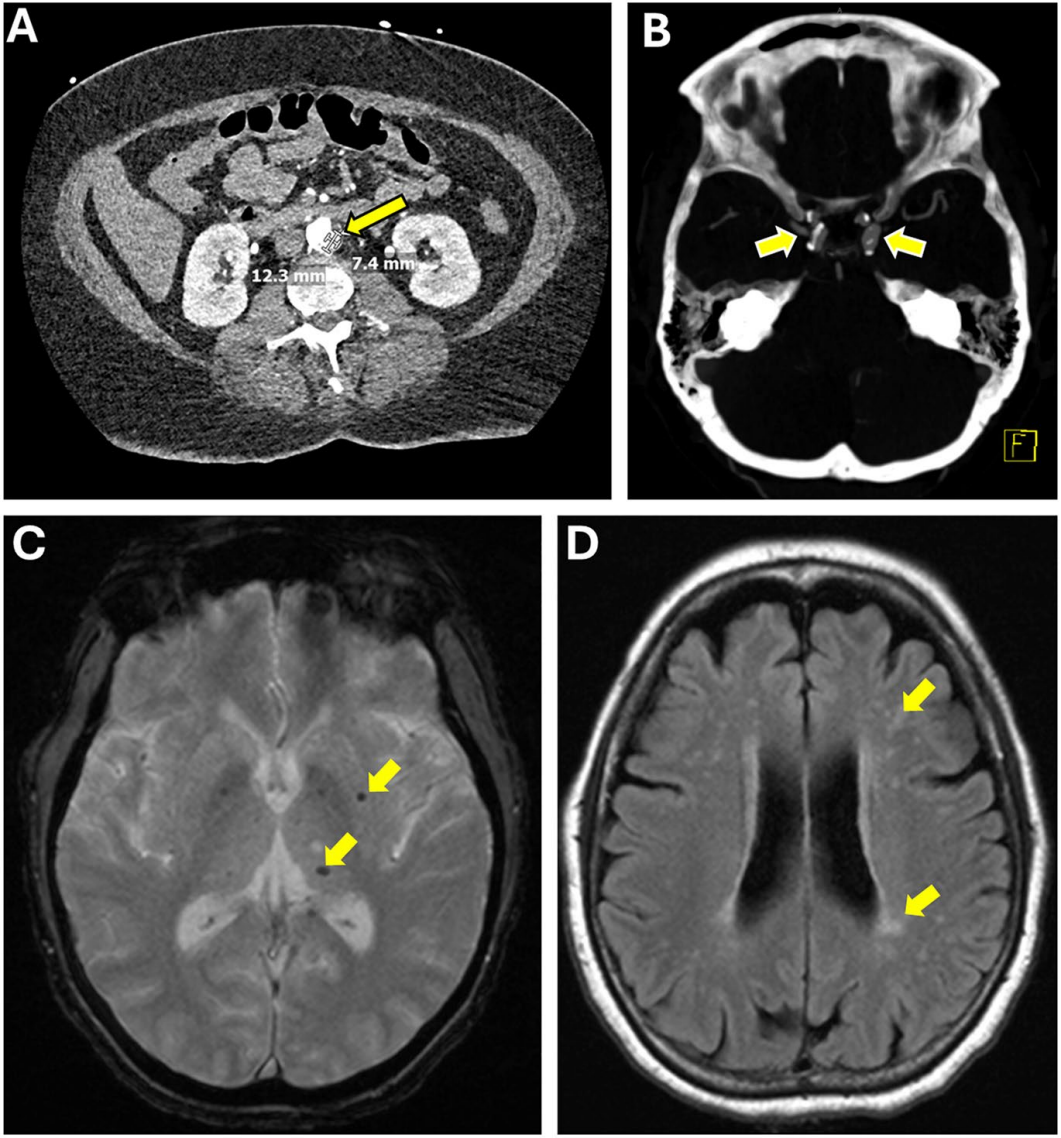
The intersection of age-related peripheral and cerebral vascular disease: a case study. This figure displays the medical journey of a 67-year-old female with a history of hypertension, non-insulin-dependent diabetes mellitus, and significant infrarenal aortic atherosclerosis (**A**, arrow) leading to severe stenosis of the left femoral artery, who experienced dizziness and nausea. Imaging revealed an acute right cerebellar infarct and right vertebral artery occlusion, suggestive of acute atherosclerosis or dissection. CT angiography further identified bilateral intracranial carotid artery stenosis due to atherosclerosis (**B**), while MRI GRE sequences showed microhemorrhages (**C**, arrowheads), and FLAIR sequences highlighted white matter hyperintensities (**D**), indicative of chronic small vessel disease. This case encapsulates the intricate connection between systemic atherosclerotic disease and its cerebral manifestations, demonstrating how atherosclerosis can precipitate both acute cerebrovascular events and chronic small vessel disease

Numerous studies have demonstrated a clear association between atherosclerosis and CSVD [44–49, 140]. Atherosclerotic vascular disease exhibits a diverse range of manifestations across various vascular territories, resulting in several clinical conditions such as carotid artery stenosis, acute myocardial infarction within coronary arteries, ischemic strokes from occlusion of intracerebral arteries, and peripheral arterial disease affecting the limbs. Clinical evidence suggests that atherosclerotic vascular disease, characterized by the build-up of plaques in large arteries in the aforementioned vascular beds, not only compromises systemic circulation but also has downstream effects on the cerebral microvasculature. Importantly, patients with atherosclerotic changes in carotid or coronary arteries are observed to have a higher prevalence of CSVD markers such as WMHs (Figs. 2, 3, and 4), CMHs, and lacunar infarcts, as revealed through neuroimaging studies [45, 46, 49, 140–149]. Estimates indicate that atherosclerosis in the peripheral circulation elevates the risk of CSVD by a factor of two to six [46, 49].

### Evidence linking peripheral atherosclerosis to BBB disruption

Recent research highlights a significant connection between peripheral atherosclerosis, BBB disruption, and the subsequent pathological outcomes resulting from compromised BBB integrity [146, 149–151]. Risk factors of atherosclerosis, including hypertension, hypercholesterolemia, hyperlipidemia, and smoking, were also reported to associate with increased BBB permeability [16, 23]. Moreover, preclinical studies indicate that atherosclerosis in large arteries [152] and carotid artery stenosis are causally associated with BBB disruption [153]. These findings, both clinical and preclinical, underscore the crucial role of vascular health in maintaining neurovascular integrity and averting a series of complications associated with BBB damage. The mechanisms underlying atherosclerosis in larger vessels—characterized by pro-inflammatory and pro-oxidative changes—mirror those affecting the cerebral circulation, highlighting the interconnected nature of systemic and cerebral vascular health.

### Shared pathomechanisms between atherosclerosis and BBB disruption

The intricate relationship between atherosclerosis and BBB disruption is underscored by shared pathomechanisms that highlight the interconnectedness of systemic vascular conditions and neurovascular health. This section explores the multifaceted roles of atherogenic diets, circulating factors—including hypercholesterolemia, inflammatory mediators, and endocrine influences—endothelial dysfunction, oxidative stress, inflammation, and endothelial senescence in contributing to both atherosclerosis and BBB integrity.

#### The role of atherogenic diets in BBB disruption: preclinical evidence

Atherogenic diets, particularly those high in fats and sugars, have been implicated in the disruption of the BBB [52, 102, 154, 155], presenting a significant link between dietary habits and neurovascular integrity. Preclinical studies utilizing animal models have provided compelling evidence on how these diets contribute to both atherosclerosis and BBB disruption, highlighting a shared pathomechanism in the progression of vascular and neurovascular diseases [51, 52, 100–102, 156–163].

High-fat diets (HFD) have been shown to induce systemic inflammation and oxidative stress, factors known to exacerbate atherosclerotic plaque formation [164, 165]. These systemic changes also affect cerebral vasculature, leading to increased BBB permeability [51, 52, 100, 101, 156–163]. Specifically, animal studies have demonstrated that prolonged exposure to HFD results in the upregulation of pro-inflammatory cytokines and MMPs and dysregulation of tight junction constituents in the brain, compromising BBB integrity [51, 102]. Importantly, the adverse effects of consumption of atherogenic diets both on BBB integrity [52] and atherogenesis [164] are exacerbated in aging, likely due to an age-related impairment of cellular oxidative stress resilience mechanisms [103, 164, 166–168].

Similarly, diets high in sugars contribute to metabolic dysregulation, including insulin resistance and hyperlipidemia, which are known risk factors for atherosclerosis. These metabolic alterations have been associated with increased BBB permeability in preclinical models [102, 163]. Moreover, the combined effects of high-fat and high-sugar diets not only amplify the risk factors for atherosclerosis but also pose a significant threat to BBB integrity [102], potentially accelerating the onset and progression of CSVD and related neurovascular complications. These preclinical findings underscore the importance of dietary habits in maintaining vascular and neurovascular health and highlight the need for further research to explore potential therapeutic interventions targeting diet-induced BBB disruption and white matter damage.

#### Circulating factors: from hypercholesterolemia and inflammatory mediators to endocrine influences

Circulating factors such as lipids, hormones, inflammatory cytokines, microRNAs, and activators of innate immunity are implicated in the pathology of both atherosclerosis and BBB disruption [45, 55, 107, 169–176, 114]. These factors can mediate vascular inflammation and endothelial damage, linking systemic vascular changes to microvascular alterations in the brain.

Preclinical studies have provided evidence that hypertriglyceridemia and hypercholesterolemia can lead to BBB disruption [55, 56, 140, 169]. This disruption is thought to stem from the direct impact of elevated triglyceride and/or cholesterol levels on the endothelial cells lining the cerebral vasculature, mirroring their detrimental effects on endothelial cells in large vessels, which are key contributors to the development of atherosclerosis [177–179].

The potential role of increased systemic levels of inflammatory cytokines during the progression of atherosclerosis is a critical area of study [180–183], given their known impact on the BBB. Inflammatory cytokines, such as tumor necrosis factor alpha (TNF-α), interleukin 1 beta (IL-1β), and interleukin 6 (IL-6), are pivotal in the inflammatory response that characterizes atherosclerosis. These cytokines contribute to endothelial dysfunction, a hallmark of atherosclerosis, by promoting the expression of adhesion molecules, attracting monocytes to the endothelium, and facilitating their transformation into foam cells within the arterial wall [180–182]. Moreover, the systemic elevation of these inflammatory mediators can also affect the cerebral vasculature, compromising the integrity of the BBB [184]. Preclinical studies confirm that systemic atherosclerosis is associated with significant cerebrovascular inflammation in mice, which is characterized by increased IL-1β [185]. Aging itself is characterized by increased levels of pro-inflammatory cytokines [186, 187]. Mounting preclinical and clinical data indicate the detrimental effect of these pro-inflammatory cytokines on BBB integrity [186–192]. Elevated levels of soluble P-selectin (sP-selectin), a biomarker for platelet/endothelial activation and a known risk factor for vascular disease, have also been shown to contribute directly to increased BBB permeability and heightened susceptibility to atherosclerosis in transgenic mouse models [193].

Apolipoprotein E (APOE), a lipid-transport protein, plays a significant role in the interplay between atherosclerosis and neurodegenerative disorders, notably dementia [194–196]. The protein's three isoforms—APOE2, APOE3, and APOE4—exhibit varied effects on lipid metabolism and neuroinflammation. APOE4, in particular, is distinguished by its association with adverse lipid profiles, increased neuroinflammation, and susceptibility to early cognitive decline and white matter damage [197–199]. This isoform significantly elevates the risk of Alzheimer's disease (AD), with heterozygotes experiencing more than twice the risk and homozygotes facing a risk increase of over ninefold [139, 197]. The risk extends to VCID, where APOE4 carriers are at a heightened risk [139]. APOE4’s influence on the BBB is profound, promoting the breakdown of essential tight junction proteins like occludin, claudin-5, and zonula occludens-1 via the low density lipoprotein receptor-related protein 1 (LRP1) signaling pathway, thus compromising BBB integrity [200]. This mechanism has been clinically correlated with BBB disruption in the limbic region among AD patients, as evidenced by MRI studies [201]. The role of APOE, especially the APOE4 allele, underscores the complex genetic factors contributing to both vascular and neurovascular pathologies.

Endocrine influences, notably the hormonal shifts accompanying aging, significantly impact vascular health [107, 112, 113, 202, 203]. A critical hormonal change is the decline in circulating levels of insulin-like growth factor 1 (IGF-1), which is essential for vascular homeostasis [202]. This decline affects both the genesis of atherosclerosis [173, 204–211] and the development of microvascular pathologies [107, 202, 212–223]. IGF-1 serves to protect the endothelium, with its age-related reduction contributing to endothelial dysfunction, a key factor in atherogenesis [221]. The decrease in IGF-1 signaling not only impairs nitric oxide production [221] and vascular oxidative stress resilience [224] but also heightens pro-oxidative [208, 225] and pro-inflammatory [205, 208, 210, 226] states within the vascular system, thereby fostering atherosclerotic plaque progression [205, 208, 210, 226]. IGF-1 confers pro-angiogenic, anti-apoptotic, and anti-senescence effects on cerebromicrovascular endothelial cells, contributing to the maintenance of the functional and structural integrity of the cerebral microcirculation [107, 202, 212–214, 217–219, 221–223, 227–230]. IGF-1 deficiency is linked to detrimental changes in cerebromicrovascular health, such as microvascular rarefaction and reduced CBF, which reflect vascular aging [218, 223]. In older adults, lower IGF-1 levels are associated with impaired neurovascular coupling (NVC) responses [212], a connection further supported by experimental models showing significant NVC impairments with induced IGF-1 deficiency [221]. Genetic disruptions in IGF-1 signaling, including IGF1R knockdown, impair endothelial and astrocytic components of NVC [214, 215] and promote BBB disruption [107], emphasizing the crucial role of IGF-1 in maintenance of neurovascular health. IGF-1 deficient mouse models also exhibit increased microvascular fragility and a higher propensity for CMHs [213, 218], with vascular wall remodeling indicating compromised structural integrity.

#### Endothelial dysfunction

Both atherosclerosis and BBB disruption share endothelial dysfunction as a fundamental pathophysiological mechanism [98, 177, 231–236]. In the context of atherosclerosis, endothelial dysfunction sets off a cascade of events leading to the formation of atherosclerotic plaques [177, 232, 234, 235]. It promotes platelet aggregation and increases vasoconstriction, further exacerbating the condition by enhancing inflammation and pathological remodeling, contributing to the narrowing and stiffening of arteries. Similarly, within the cerebral vasculature, endothelial dysfunction significantly impacts BBB integrity [43].

#### Oxidative stress and inflammation

Oxidative stress and inflammation are critical factors in the progression of both atherosclerosis and CSVD, acting as intertwined pathological processes [33, 41–43]. The oxidative modification of lipoproteins and endothelial activation in atherosclerosis and the oxidative damage to cerebral endothelial cells in CSVD are examples of how oxidative stress serves as a common pathogenic pathway. Similar to the alterations seen in larger vessels during aging, the microcirculation experiences an increased cellular production of reactive oxygen species (ROS) [237–244]. This escalation in ROS production is primarily driven by age-related factors, such as a decline in cellular Nicotinamide adenine dinucleotide (NAD^+^) levels, dysregulation of sirtuin 1 (SIRT1), and a consequent increase in mitochondrial ROS generation [237, 240]. This heightened state of microvascular oxidative stress is a crucial contributor to endothelial dysfunction [237, 240], a central aspect of CSVD. It impairs the bioavailability of nitric oxide and fosters inflammatory responses, which are critical in the pathogenesis of CSVD [34, 231, 245, 246]. The activation of endothelial cells, marked by the expression of adhesion molecules and recruitment of inflammatory cells, also increases the propensity for thrombosis. These changes within the cerebral microcirculation have direct implications for several key pathological manifestations of CSVD. Oxidative stress and endothelial dysfunction are intimately associated with the disruption of the BBB [52, 247], the impairment of neurovascular coupling responses [237, 240, 241], formation of lacunar infarcts, and the pathogenesis of microvascular fragility and CMHs [244].

In the realm of inflammation, robust preclinical and clinical evidence indicates that systemic inflammation is a critical trigger for BBB disruption. The underlying mechanisms are complex and involve various circulating inflammatory mediators, including cytokines. Cellular components of the BBB, such as endothelial cells, pericytes, and astrocytes, are key players in this process. They express pattern recognition receptors and activate inflammasomes in response to blood-borne signals, including Pathogen-Associated Molecular Patterns (PAMPs) and Damage-Associated Molecular Patterns (DAMPs) [190, 191]. This activation leads to increased BBB permeability and the secretion of proinflammatory cytokines and chemokines like IL-1β, IL-1α, IL-6, monocyte chemoattractant protein-1 (MCP-1), and C-C motif chemokine ligand 5 (CCL5, also known as RANTES), which further exacerbate BBB disruption and promote leukocyte attraction, creating a self-perpetuating cycle of inflammation [189, 192].

There is strong preclinical and clinical evidence that systemic inflammation triggers BBB disruption [248, 249]. Chronic low-grade systemic inflammation associated with systemic atherosclerosis can also trigger these BBB-disrupting mechanisms. There is evidence that an elevated inflammatory status within atherosclerotic plaques is associated with increased BBB disruption and neuroinflammation [250, 251]. Therefore, chronic inflammatory states, as seen in peripheral atherosclerosis, might play a crucial role in BBB dysfunction. This relationship highlights the systemic nature of these pathologies, underscoring the need for a comprehensive approach to understanding and managing the interaction between peripheral atherosclerosis and cerebral microvascular health.

#### Endothelial senescence

As individuals age, there is a marked increase in the number of cells entering a state of senescence primarily due to the accumulation of oxidative stress-induced DNA damage [172, 252–259]. The accumulation of senescent cells is particularly prominent within atherosclerotic plaques, a phenomenon that is further accelerated by common cardiovascular risk factors such as hypertension and diabetes mellitus [172, 252, 254, 255, 260–267]. Within the vascular system, these senescent cells are known to secrete a range of pro-inflammatory and matrix-degrading molecules, collectively referred to as the senescence-associated secretory phenotype (SASP) [41, 42]. SASP factors contribute to chronic inflammation and can lead to increased plaque instability in atherosclerotic vascular diseases. Additionally, the shortening of telomeres in vascular cells is another factor that drives cellular senescence, further influencing the formation and progression of atherosclerotic plaques.

Accumulation of senescent cells is also crucial aspect of microvascular aging and pathology, especially in the context of CSVD [50, 107, 268, 269]. Oxidative DNA damage-mediated senescence is particularly pronounced in cerebromicrovascular endothelial cells [108, 269, 270]. As microvascular endothelial cells become senescent, they exhibit a SASP, characterized by the secretion of various pro-inflammatory cytokines and matrix-degrading enzymes [271]. This molecular cascade significantly exacerbates microvascular damage and plays a key role in the development of CSVD [106, 108, 268, 270, 272]. These findings underscore the intricate link between cellular aging processes and the development of both macrovascular and microvascular pathologies, highlighting the importance of understanding cellular senescence in the context of the continuum of accelerated vascular aging.

In recent years, research has shed light on the potential for rejuvenating the cerebral microcirculation by specifically targeting senescent cells [106, 108, 268, 270]. Studies employing pharmacological or genetic interventions to eliminate these cells in aging mouse models have yielded encouraging results. These outcomes include the restoration of endothelial function, improvements in neurovascular coupling responses, and bolstered integrity of the BBB [106, 108, 268, 270]. A notable example is the use of the BCL-2 inhibitor senolytic drug Navitoclax, which has been shown to reverse both BBB disruption [106] and mitigate the development of CMHs induced by hypertension in aged mice [268]. This discovery is particularly significant as it points towards a promising therapeutic approach to prevent microvascular changes that are instrumental in the pathogenesis of CSVD associated with systemic atherosclerosis. By targeting the senescent cells that contribute to accelerated microvascular aging and dysfunction, there is potential to address a fundamental aspect of CSVD and perhaps ameliorate its impact on brain health.

In summary, understanding the continuum of vascular aging, which links systemic and cerebral vascular health, is crucial. Recognizing the shared pathomechanisms between peripheral atherosclerosis and BBB disruption is essential for developing comprehensive strategies to mitigate CSVD progression, particularly in older adults with increased cardiovascular risk.

## Clinical implications of BBB disruption in atherosclerosis and CSVD

Progressive BBB disruption facilitates the entry of neurotoxic substances, inflammatory cytokines, and cells into the brain parenchyma, contributing to neuroinflammation, neuronal damage, synaptic dysfunction, and, ultimately, cognitive decline [24, 273, 274]. It is likely that compromised BBB integrity and its sequalae are closely linked to the development of a spectrum of cognitive impairments ranging from mild cognitive deficits to severe dementia [24, 25]. The progression from age-related BBB disruption in the context of CSVD and atherosclerosis to cognitive impairment underscores the critical role of vascular health in maintaining cognitive function [24, 37, 132, 273–276].

Early identification of individuals at risk of BBB disruption and subsequent cognitive decline is essential for preventing or mitigating the impact of VCID [116, 274]. Risk assessment strategies may include the evaluation of vascular risk factors such as hypertension, diabetes mellitus, and hyperlipidemia, alongside the use of advanced neuroimaging techniques to detect early signs of BBB permeability in patients with peripheral atherosclerotic diseases. Biomarkers in blood and cerebrospinal fluid, reflecting endothelial dysfunction and BBB integrity may also hold promise for early detection and monitoring of disease progression.

Protecting or restoring BBB integrity offers a promising avenue for future therapeutic intervention. Targeting the underlying mechanisms of BBB disruption, such as inflammation, oxidative stress, and endothelial cell dysfunction, could mitigate the progression of VCID in high risk patients. Additionally, emerging research on senolytics and drugs that target specific pathways implicated in BBB disruption (e.g., the signaling pathways involving APOE4) highlights the potential for novel interventions [273]. Modulating lifestyle factors, such as consumption of a healthy diet and regular exercise, which have been shown to influence vascular health and BBB integrity, also presents a viable strategy for protecting cognitive function [277, 278].

## Conclusion

In conclusion, the evidence compiled from both clinical and preclinical studies unequivocally underscores the intricate connection between atherosclerotic vascular diseases in the peripheral circulation and BBB disruption, shedding light on the significant impact of vascular health on neurovascular integrity. The findings reveal that risk factors commonly associated with atherosclerosis, such as hypertension, hypercholesterolemia, hyperlipidemia, and smoking, are also implicated in the pathogenesis of CSVD and increased BBB permeability, which, in turn, can lead to a cascade of neurocognitive consequences. This research highlights the critical role that systemic vascular health plays in preserving the BBB and, by extension, in preventing the myriad complications that stem from its breakdown. The shared mechanisms of pro-geronic, pro-inflammatory, and pro-oxidative changes across the vascular system emphasize the need for holistic approaches in managing vascular health to safeguard against CSVD and VCID. Moving forward, it is imperative that future research continues to explore the relationship between vascular pathology and BBB integrity to develop targeted interventions that can mitigate the risk of CSVD and maintain cognitive function in aging populations.
